# Short-term postoperative neurocognitive safety and recovery profiles of remimazolam tosilate combined with flumazenil vs. propofol in cirrhotic patients undergoing endoscopic variceal treatment: a randomized controlled trial

**DOI:** 10.3389/fmed.2026.1871958

**Published:** 2026-09-04

**Authors:** Yanjie Chen, Xiangrong Du, Fu Shi, Na Zhu

**Affiliations:** 1 Department of Anesthesiology, Liaocheng Anesthesia and Brain Function Laboratory, Liaocheng People’s Hospital, Liaocheng, Shandong, China; 2 Department of General Medicine, Liaocheng People’s Hospital, Liaocheng, Shandong, China

**Keywords:** endoscopic treatment, esophagogastric varices, flumazenil, liver cirrhosis, neurocognitive function, propofol, quality of recovery, remimazolam tosilate

## Abstract

**Background:**

Patients with liver cirrhosis frequently exhibit compromised drug metabolism due to hepatic dysfunction, which significantly increases their susceptibility to postoperative neurological adverse events. This study aimed to evaluate the impact of remimazolam tosilate combined with flumazenil on early postoperative neurocognitive function and recovery quality in cirrhotic patients undergoing endoscopic variceal ligation (EVL) and/or endoscopic variceal sclerotherapy (EVS), so as to provide evidence-based guidance for optimizing clinical anesthesia management.

**Methods:**

A total of 108 cirrhotic patients scheduled for EVL and/or EVS were randomly assigned to the propofol group (Group P, *n* = 54) or the remimazolam tosilate group (Group R, *n* = 54). The primary outcomes were defined as performance on the Number Connection Test-A (NCT-A) and the Digit Symbol Test (DST), evaluated at baseline (preoperatively) and at 6 and 24 h following surgery. Secondary endpoints included the time to spontaneous respiration, time to eye opening, time to commanded response, and time to extubation. Furthermore, the modified Observer's Assessment of Alertness/Sedation (MOAA/S) scores and modified Steward scores were assessed at multiple post-extubation time points.

**Results:**

No statistically significant differences were observed in NCT-A and DST scores between the two groups at baseline, 6 h postoperatively, or 24 h postoperatively (*P* > 0.05). Relative to preoperative baseline levels, the NCT-A completion time was significantly reduced, while DST scores were significantly increased at 24 h postoperatively in both groups (*P* < 0.05). Regarding the quality of recovery, the times to spontaneous respiration, eye opening, commanded response, and extubation were significantly shorter in Group R compared to Group P (*P* < 0.01). Furthermore, at the time of extubation (T0), the modified MOAA/S and Steward scores were significantly higher in Group R compared to Group P (*P* < 0.05).

**Conclusion:**

In cirrhotic patients undergoing endoscopic variceal ligation or sclerotherapy, the administration of remimazolam tosilate with flumazenil demonstrated comparable early postoperative neurocognitive performance to propofol, while significantly shortening recovery time and enhancing the quality of extubation and emergence. Consequently, this regimen represents a neurologically safe (with regard to short-term cognitive outcomes), rapidly controllable, and clinically feasible anesthetic option for this high-risk population.

**Clinical trials registration:**

this study was registered on https://www.chictr.org.cn, (ChiCTR2200066161).

## 1 Introduction

Esophagogastric variceal bleeding is one of the most common and severe complications of cirrhotic portal hypertension, characterized by a high risk of rupture and hemorrhage, as well as poor prognosis (1). Endoscopic variceal ligation (EVL) and endoscopic variceal sclerotherapy (EVS) have become first-line treatments for esophagogastric varices, owing to their proven efficacy and minimal invasiveness (2, 3). Patients with cirrhosis typically require endoscopic procedures under anesthesia. Currently, multiple anesthesia protocols are available; however, selecting appropriate sedative agents for patients with cirrhosis remains a significant challenge.

Our team's previous study also confirmed that performing EVL and/or EVS under endotracheal intubation and general anesthesia provides more stable conditions for endoscopic procedures and effectively reduces the risk of perioperative reflux aspiration (4). However, cirrhosis leads to reduced activity of drug-metabolizing enzymes, decreased hepatic blood flow, and diminished plasma protein binding capacity. These alterations result in a prolonged elimination half-life of various sedative and anesthetic agents, increasing the risk of drug accumulation. Consequently, cirrhotic patients are more susceptible to delayed emergence, respiratory depression, and the induction or exacerbation of hepatic encephalopathy, which can adversely affect postoperative neurological recovery and early rehabilitation (5, 6). Midazolam is a commonly utilized sedative for endoscopy and is primarily metabolized by the liver. In patients with liver cirrhosis, midazolam usage may lead to prolonged recovery time, delayed recovery of neuropsychiatric function due to accumulation of active metabolites, and induction of hepatic encephalopathy (7, 8). Minimal hepatic encephalopathy (MHE) is an early and subtle manifestation of neurocognitive impairment in patients with cirrhosis, characterized by deficits in specific cognitive domains such as attention, executive function, and visuomotor coordination (9). The Number Connection Test-A (NCT-A) and the Digit Symbol Test (DST) are easy to administer and exhibit favorable specificity. They serve as sensitive tools for screening MHE and dynamically monitoring perioperative neurocognitive function, and can be used to objectively evaluate the effects of anesthetic agents on central nervous system function (10, 11).

Propofol is currently a widely used intravenous anesthetic for endoscopic procedures in patients with cirrhosis, owing to its rapid onset, short duration of action, and relatively favorable recovery profile compared to traditional benzodiazepines such as midazolam (12–14). However, the metabolism of propofol still mainly depends on the liver, and it produces significant dose-dependent depressant effects on the cardiovascular and respiratory systems, which requires extra caution in patients with poor hepatic functional reserve and hemodynamic instability (15). Consequently, propofol represents a clinically relevant benchmark against which novel anesthetic strategies—such as the remimazolam-flumazenil combination—should be compared. A head-to-head comparison between these two approaches is essential to determine whether the theoretical pharmacological advantages of remimazolam translate into tangible clinical benefits in this high-risk population. As a novel ultra-short-acting benzodiazepine, remimazolam tosylate is metabolized not via hepatic or renal function, but through rapid hydrolysis by tissue non-specific esterases, with no accumulation during continuous infusion; moreover, its effect can be rapidly reversed by its specific antagonist flumazenil (16, 17). Flumazenil, a specific competitive antagonist at benzodiazepine receptors, plays an essential role in this combined strategy. It rapidly reverses the sedative effects of remimazolam by displacing the drug from its receptor binding sites, thereby accelerating emergence and reducing the duration of postoperative sedation (18). In cirrhotic patients, who are at increased risk of prolonged sedation and hepatic encephalopathy, flumazenil antagonism may offer the additional benefit of minimizing residual benzodiazepine effects and facilitating early neurological recovery (19). Therefore, the remimazolam-flumazenil combination represents a pharmacologically distinct strategy: remimazolam provides rapid, esterase-based metabolism independent of hepatic function, while flumazenil offers a specific “off-switch” that can be titrated to achieve timely emergence. This dual mechanism may confer advantages over propofol, which lacks a specific pharmacological antagonist and relies entirely on redistribution and hepatic clearance for recovery.

A prior study conducted by our group demonstrated that remimazolam-based general anesthesia maintains favorable hemodynamic stability during pituitary adenoma resection and reduces the incidence of hypotension, thereby mitigating, to a certain extent, the risks associated with prolonged sedation (20). Prior studies have also shown that remimazolam is safe and effective when used for painless gastroscopy and EVL procedures in patients with liver cirrhosis (4, 21). Nevertheless, current evidence lacks systematic comparisons between the remimazolam-flumazenil regimen and propofol regarding short-term postoperative neurocognitive function and quality of recovery in patients with cirrhosis undergoing endoscopic variceal ligation or sclerotherapy.

Accordingly, we designed a prospective, randomized, controlled trial to systematically evaluate the effects of remimazolam tosilate combined with flumazenil vs. propofol on early postoperative neurocognitive function (assessed using the NCT-A and DST) and recovery quality in cirrhotic patients undergoing EVL and/or EVS. The findings are expected to provide a reference for optimizing clinical anesthesia management strategies in this specific patient population.

## 2 Materials and methods

### 2.1 Study design

This work was a prospective, single-center, randomized, single-blind, controlled trial comparing remimazolam and propofol for the quality of recovery after endoscopic treatment in cirrhotic patients. The study was approved by the Ethics Committee of Liaocheng People's Hospital, Shandong Province, China (Approval No. 2022266) and was conducted in accordance with the Declaration of Helsinki. The trial was registered with the Chinese Clinical Trial Registry (ChiCTR2200066161) and filed in the Medical Research Registration Information System (Filing No. MR-37-23-006728). All patients or their legal guardians provided written informed consent. Participants were assigned sequentially according to the order of enrollment and then allocated to either the propofol group (Group P) or the remimazolam group (Group R) based on the randomization numbers from a computer-generated randomization code table. The subjects were blinded to the anesthetic regimen and the choice of anesthetic drugs. Anesthesiologists administered the anesthesia according to the group allocation. All patients, operating physicians, and data collectors involved in this study were blinded to the randomization assignment.

### 2.2 Study population

Patients with liver cirrhosis who underwent EVL and/or EVS at our hospital from December 2022 to December 2024 were enrolled as study subjects. Inclusion criteria: ① Patients aged 18 to 65 years; ② Patients with liver cirrhosis who met the criteria for endoscopic EVL and/or EVS; ③ Esophageal varices morphology grade G Ⅱ to G Ⅲ; ④ Liver function Child-Pugh class A, B, or C; ⑤ Platelet count >50 × 10^9^/L; ⑥ Variceal diameter 0.3–2 cm; ⑦ Patients who agreed to undergo endoscopic treatment and signed informed consent. Exclusion criteria: ① American Society of Anesthesiologists (ASA) physical status IV or V; ② Addiction to alcohol, opioids, or sedative-hypnotic drugs; ③ Overt hepatic encephalopathy, psychiatric illness, mental disorder, or active neurological disorder; ④ Neurological diseases such as Alzheimer's disease or Parkinson's disease; ⑤ Perioperative hemorrhagic shock; ⑥ Known allergy to protein products, tofu, soy, propofol, or benzodiazepines.

### 2.3 Anesthesia protocol

After entering the operating room, an intravenous line was established, and oxygen was administered via a face mask. Electrocardiogram (ECG), heart rate (HR), mean arterial pressure (MAP), pulse oxygen saturation (SpO_2_), end-tidal carbon dioxide partial pressure (PetCO_2_), and bispectral index (BIS) were monitored. The anesthesia protocol for all patients followed the routine procedure of our center, and all procedures were performed by physicians with more than five years of clinical experience.

Awake endotracheal intubation with general anesthesia was used in all patients as per our institutional standard protocol for endoscopic variceal treatment. This approach was selected to effectively prevent the risk of perioperative regurgitation and aspiration, which is of particular concern in cirrhotic patients with esophageal varices due to the presence of portal hypertension, potential gastric stasis, and the risk of bleeding during instrumentation. The awake intubation procedure was as follows: 5 mL of 2% lidocaine was sprayed onto the anterior part of the tongue to achieve topical anesthesia of the tongue, tongue base, and pharynx in a downstream manner. Fentanyl 3 µg/kg was slowly injected intravenously. Three minutes later, 3 mL of lidocaine was sprayed through a laryngotracheal anesthesia cannula for subglottic topical anesthesia. After two minutes, tracheal intubation was performed using a video laryngoscope. After successful awake tracheal intubation, anesthesia induction was carried out. In the propofol group (Group P), the induction dose was 2 mg/kg intravenously. In the remimazolam group (Group R), the induction dose was 0.2 mg/kg intravenously.

The maintenance dose of propofol was 4–8 mg/kg/h by intravenous infusion in Group P, and the maintenance dose of remimazolam was 1–2 mg/kg/h by intravenous infusion in Group R. Remifentanil was infused at a dose of 0.1–0.2 µg/kg/min. During the procedure, tidal volume was maintained at 6–8 mL/kg, respiratory rate at 12–14 breaths/min, and PetCO_2_ at 35–45 mmHg. BIS was maintained between 50 and 60, and blood pressure and heart rate were kept within 20% of baseline values. If hypotension occurred (systolic blood pressure <30% of baseline or MAP <60 mmHg), ephedrine (0.1 mg/kg, IV) was administered. If bradycardia occurred (HR < 50 bpm), atropine (0.3 mg, IV) was given.

At the end of the procedure, all infusions were stopped. Patients in Group R received a slow intravenous injection of 0.5 mg flumazenil, while those in Group P received the same volume of normal saline. When patients regained consciousness, resumed regular spontaneous breathing with a tidal volume greater than 6 mL/kg, and had stable vital signs, the tracheal tube was removed. The patients were then transferred to the post-anesthesia care unit (PACU) for observation and returned to the ward when the discharge criteria were met.

### 2.4 Endoscopic procedure

Endoscopic tissue adhesive injection combined with sclerotherapy and ligation was performed according to our institutional standard protocol. After successful anesthesia and airway management, a gastroscope was inserted to identify gastric fundus and cardia varices and to select the optimal injection site. The modified sandwich technique (2 mL of 1% lauromacrogol—1 mL of tissue adhesive—2 mL of 1% lauromacrogol) was used for tissue adhesive injection. Subsequently, esophageal varices were treated with endoscopic variceal ligation using a six-shot ligator (Cook Medical), with intensive ligation performed in a spiral manner from the cardia upward, targeting all visible varices with more than seven ligation points per session and approximately 1.5 cm spacing between sites.

### 2.5 Outcome measures

#### 2.5.1 Primary outcome measure: neuropsychological tests

The NCT-A and DST were administered to patients before operation, and at 6 h and 24 h after operation. These tests have been validated as sensitive and specific tools for screening minimal hepatic encephalopathy and assessing perioperative neurocognitive function (12, 22). The tests were performed by trained researchers in a quiet environment free from external distractions.

NCT-A refers to the time required for the patient to connect randomly arranged Arabic numerals 1 through 25 in ascending order as quickly as possible. The patient was informed that the pen tip should not leave the paper during the connection process, and errors were corrected promptly; the recorded time included error correction. Abnormal values were defined as the mean plus two standard deviations. After age adjustment, diagnostic criteria were: >34.3 s in patients aged <35 years; >45.7 s in patients aged 35 to 44 years; >52.8 s in patients aged 45 to 54 years, and >61.9 s in patients aged >55 years.

In the DST test, numbers 1 to 9 are each assigned a different symbol, and the subject is required to fill in the corresponding symbol under each number as quickly and accurately as possible within 90 s across a total of 90 blanks. Scoring is as follows: a correctly written symbol counts 1 point, an inverted symbol counts 0.5 points, and a completely wrong symbol counts 0 points; the total score within 90 s is then calculated. Abnormal values are defined as the mean minus two standard deviations. After age adjustment, diagnostic criteria were: <40.5 in patients <35 years; <35 in patients aged 35 to 44 years; <28.5 in patients aged 45 to 54 years and <26 in patients aged >55 years.

#### 2.5.2 Secondary outcome measures: indicators of recovery quality

(a) The following times were recorded for both groups: spontaneous respiration time (from the end of the procedure to the return of spontaneous respiration), eye opening time (from the end of the procedure to the time when the patient could open their eyes on command), commanded response time (from the end of the procedure to the time when the patient could perform a command-directed action, such as hand squeezing), and extubation time (from the end of the procedure to the final removal of the tracheal tube).
(b) The modified Observer's Assessment of Alertness/Sedation (MOAA/S) score and the modified Steward score were used to evaluate the quality of recovery immediately after extubation (T0) and at 5 min (T1), 10 min (T2), 15 min (T3), and 20 min (T4) after extubation. The scoring criteria are as follows:

Modified MOAA/s score (assesses depth of sedation): 5 points, responds readily to normal tone of speech; 4 points, responds sluggishly to normal tone of speech; 3 points, responds only after loud or repeated calling; 2 points, responds only to mild prodding or shaking; 1 point, responds only to painful stimulus (trapezius squeeze); 0 points, no response to trapezius squeeze.

Modified Steward score includes three components: ① Level of consciousness: fully awake (2 points), responsive to stimuli (1 point), unresponsive to stimuli (0 points); ② Airway patency: can cough on command (2 points), maintains airway without support (1 point), requires airway support (0 points); ③ Limb mobility: purposeful movement (2 points), non-purposeful movement (1 point), no movement (0 points). The total score ranges from 0 to 6 points, and a score of ≥4 points is considered the discharge criterion from the PACU.

### 2.6 Sample size calculation

Based on the pre-experimental results, the NCT-A score in patients after propofol anesthesia at 6 h post-operation was 60.1 (standard deviation, 22), and the NCT-A score in patients after remimazolam anesthesia at 6 h post-operation was 59 (standard deviation, 22). A non-inferiority study was designed using PASS 15.0 software with a non-inferiority margin (*δ*) set at 12.02 (20% of the NCT-A score in patients after propofol anesthesia at 6 h post-surgery). The sample size was calculated with *α* = 0.05 and *β* = 0.1, resulting in 49 patients required per group. Considering a 10% dropout rate, the final sample size was set at 54 patients per group, giving a total sample size of 108 patients. Eligible patients were randomly assigned to two groups using a random number table: the propofol group (Group P, *n* = 54) and the remimazolam tosilate group (Group R, *n* = 54).

### 2.7 Statistical analysis

Statistical analysis was performed using SPSS 21.0 software. Normally distributed continuous data were expressed as mean ± standard deviation [(x¯) ± s]. Differences between the two groups were tested using the independent samples *t*-test, and comparisons among different time points within the same group were analyzed using repeated measures analysis of variance. Non-normally distributed continuous data were expressed as median with interquartile range and compared using non-parametric tests; between-group comparisons were performed using the rank sum test. Categorical data were described as frequencies and percentages and compared using the chi-square test. *P* value <0.05 was considered statistically significant.

## 3 Results

**Figure 1:**
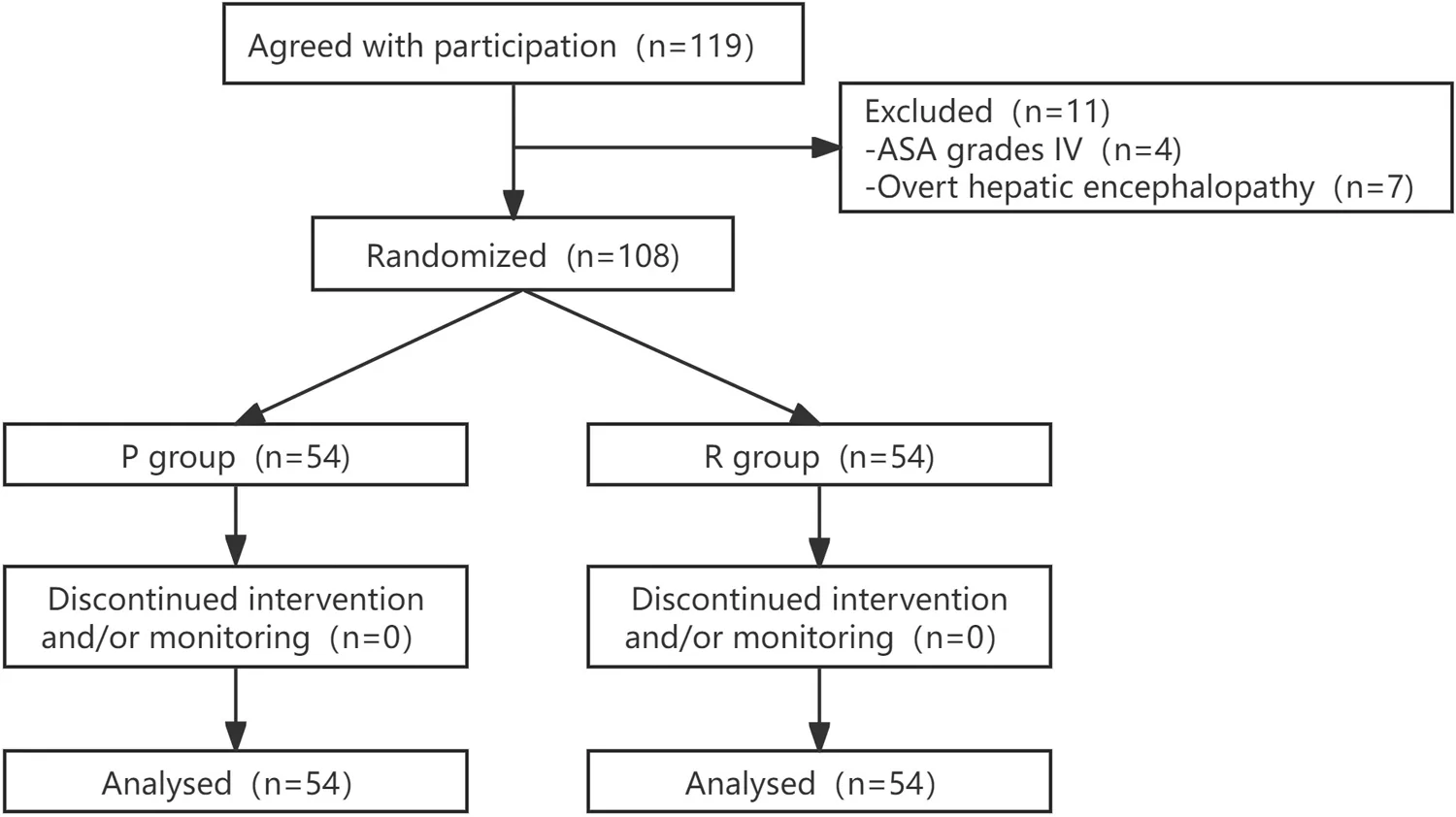
Flow diagram of patients. ASA: American Society of Anesthesiologists.

A total of 119 patients were initially enrolled in this study. Among them, four patients were excluded due to ASA physical status class IV, and seven due to overt hepatic encephalopathy. Finally, 108 patients were randomized (54 in each group) and included in the final analysis. No patients were lost to follow-up, as shown in Figure 1.

### 3.1 Baseline patient characteristics

There were no statistically significant differences between the two groups in baseline characteristics, including sex, etiology, clinical symptoms, Child-Pugh classification, educational level, incidence of ascites, height, weight, body mass index (BMI), age, liver and kidney function, coagulation profile, surgical duration, or postoperative hospital stay (*P* > 0.05), as shown in Table 1.

**Table 1:** Baseline patient characteristics of the two groups.

Item	Group P (*n* = 54)	Group R (*n* = 54)	*P-*value
Age (year)	55.08 ± 10.47	54.20 ± 9.62	0.655
Sex (M/F)	36/18	28/26	0.117
Height (cm)	166.54 ± 7.17	166.48 ± 7.30	0.968
Weight (kg)	66.68 ± 10.56	64.25 ± 12.48	0.276
BMI (kg cm^−2^)	23.99 ± 3.21	23.07 ± 3.67	0.173
Child-pugh scale/ (*n*, %)			0.065
A	30 (55.56)	18 (33.33)	
B	20 (37.04)	29 (53.70)	
C	4 (7.41)	7 (12.96)	
Symptom (*n*, %)			0.634
Haematemesis	12 (22.22)	16 (29.63)	
Melena	28 (51.85)	25 (46.30)	
Reexamination	3 (5.56)	5 (9.26)	
Others	11 (20.37)	8 (14.81)	
Pathogeny (*n*, %)			0.535
HBV	34 (62.96)	28 (51.85)	
HCV	1 (1.85)	3 (5.56)	
Alcoholic hepatitis	8 (14.81)	7 (12.96)	
Liver cancer	1 (1.85)	1 (1.85)	
Others	10 (18.52)	15 (27.78)	
Education status (*n*, %)			0.117
Primary school	16 (29.63)	26 (48. 15)	
Junior high school	9 (16.67)	10 (18.52)	
Senior high school	23 (42.59)	12 (22.22)	
University education	6 (11. 11)	6 (11. 11)	
Laboratory examination			
Aspartate aminotransferase	48.85 ± 58.02	37.39 ± 21.49	0.176
Alanine aminotransferase	33.20 ± 21.22	35.43 ± 34.59	0.688
Bilirubin (μmol/L)	28.09 ± 27.20	27. 12 ± 15.21	0.818
Albumin (g/L)	32.65 ± 5.76	32.76 ± 5.47	0.918
Hemoglobin (g/L)	88.00 ± 21.88	88.57 ± 18.49	0.883
WBC (×109/L)	4.66 ± 2.89	5.38 ± 7.46	0.507
Platelet (×109/L)	81.67 ± 59.95	100.87 ± 70.64	0.132
Creatinine (μmol/L)	60.57 ± 15.39	62.82 ± 18.99	0.501
Prothrombin time (s)	15.27 ± 2.30	14.92 ± 2.31	0.430
Surgery time (min)	29.35 ± 8.81	28.22 ± 7.17	0.466
Length of stay (day)	8.85 ± 2.91	8. 13 ± 3.10	0.214

Variables presented as mean ± standard deviation [(x¯) ± s] or number of patients *n* (%). BMI: body mass index, HBV: hepatitis B virus, HCV: hepatitis C virus, WBC: White Blood Cell.

According to the results in Table 1, hepatitis was the primary etiology of liver cirrhosis. The main clinical manifestations included hematemesis and/or melena, with Child-Pugh classifications predominantly being grades A and B. Additionally, cirrhosis was commonly associated with complications such as hypoalbuminemia, anemia, and abnormal liver function.

### 3.2 Primary outcome measures

#### 3.2.1 Comparison of NCT-A and DST scores between the two groups

Comparisons between groups revealed no statistically significant differences in neurological safety, as assessed by changes in NCT-A and DST scores at the preoperative, 6 h postoperative, and 24 h postoperative time points (*P* > 0.05).

**Figure 2:**
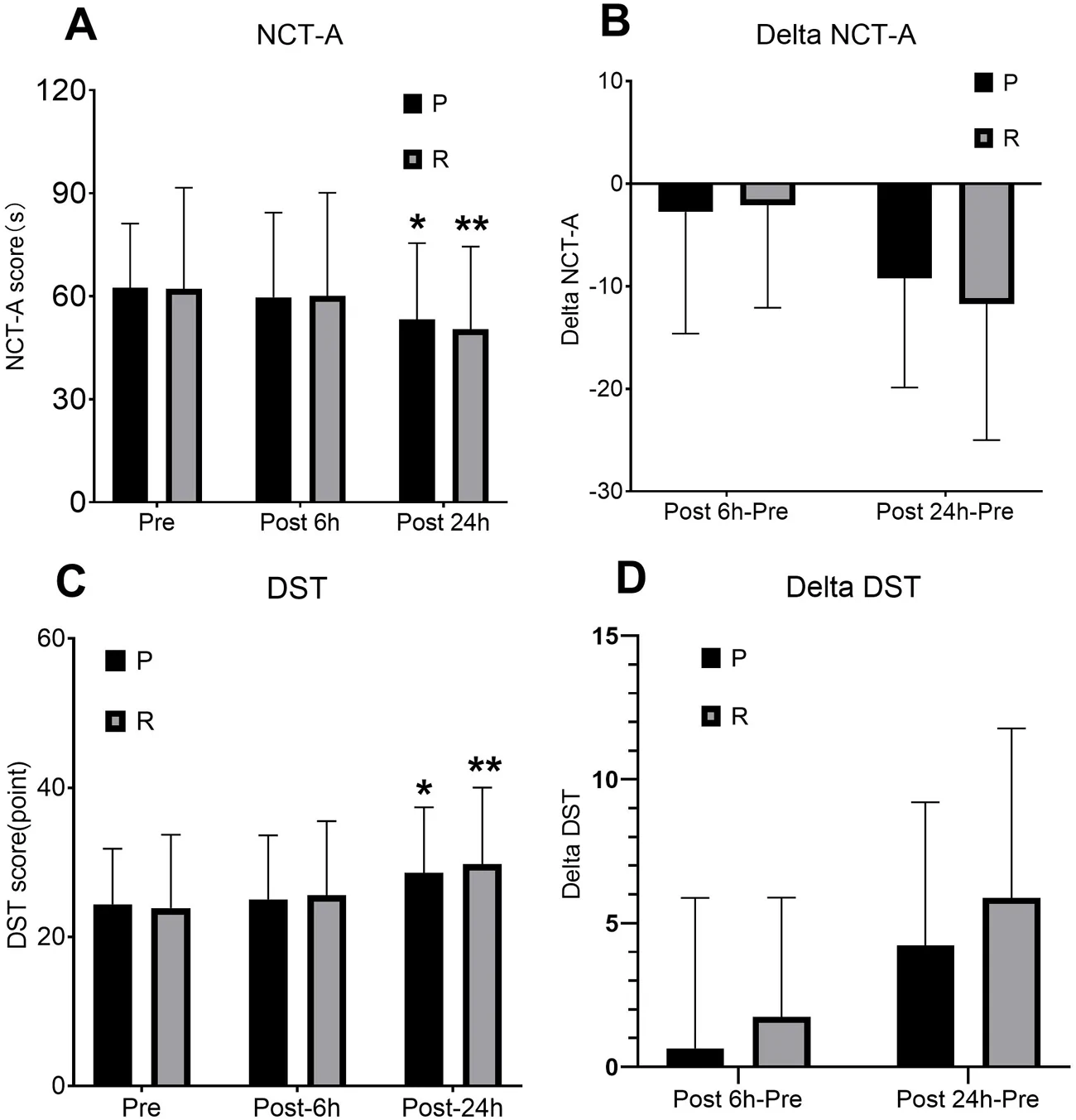
Comparisons of NCT-A and DST scores at different time point between the two groups. **(A)** NCT-A completion time at pre-, 6 h, 24 h postoperatively; **(B)** Delta NCT-A (Post-Pre) at 6 h and 24 h; **(C)** DST scores at pre-, 6 h, 24 h postoperatively; **(D)** Delta DST (Post-Pre) at 6 h and 24 h. Comparisons of NCT-A and DST scores between Group P and Group R at baseline, 6 h, and 24 h postoperatively. Data are presented as mean ± standard deviation (SD). ★ *P* < 0.05 vs. baseline; ★★ *P* < 0.01 vs. baseline. NCT-A, number connection test-A; DST, digit symbol test.

Intra-group analyses demonstrated a significant reduction in NCT-A scores and a corresponding increase in DST scores in both groups at 24 h postoperatively, as displayed in Figures 2A,C. In Group P, the NCT-A score at 24 h post-operation (53.21 ± 22.26 s) was significantly lower compared to the preoperative baseline (62.45 ± 18.72 s, *P* = 0.031), whereas the DST score at the same time point (28.62 ± 8.76 points) was significantly elevated relative to the baseline value (24.38 ± 7.46 points, *P* = 0.009). In Group R, the NCT-A score at 24 h post-operation (50.47 ± 24.01 s) was significantly lower compared to the preoperative baseline (62.21 ± 29.37 s, *P* = 0.028), whereas the DST score at the same time point (29.75 ± 10.28 points) was significantly elevated relative to the baseline value(23.87 ± 9.84 points, *P* = 0.002).

In addition, we also compared the delta values at 6 h and 24 h postoperatively relative to baseline value between the two groups, respectively. The differences were not statistically significant (*P* > 0.05), as seen in Figures 2B,D.

#### 3.2.2 Non-inferiority comparison at 6 h post-operation between the two groups

**Figure 3:**
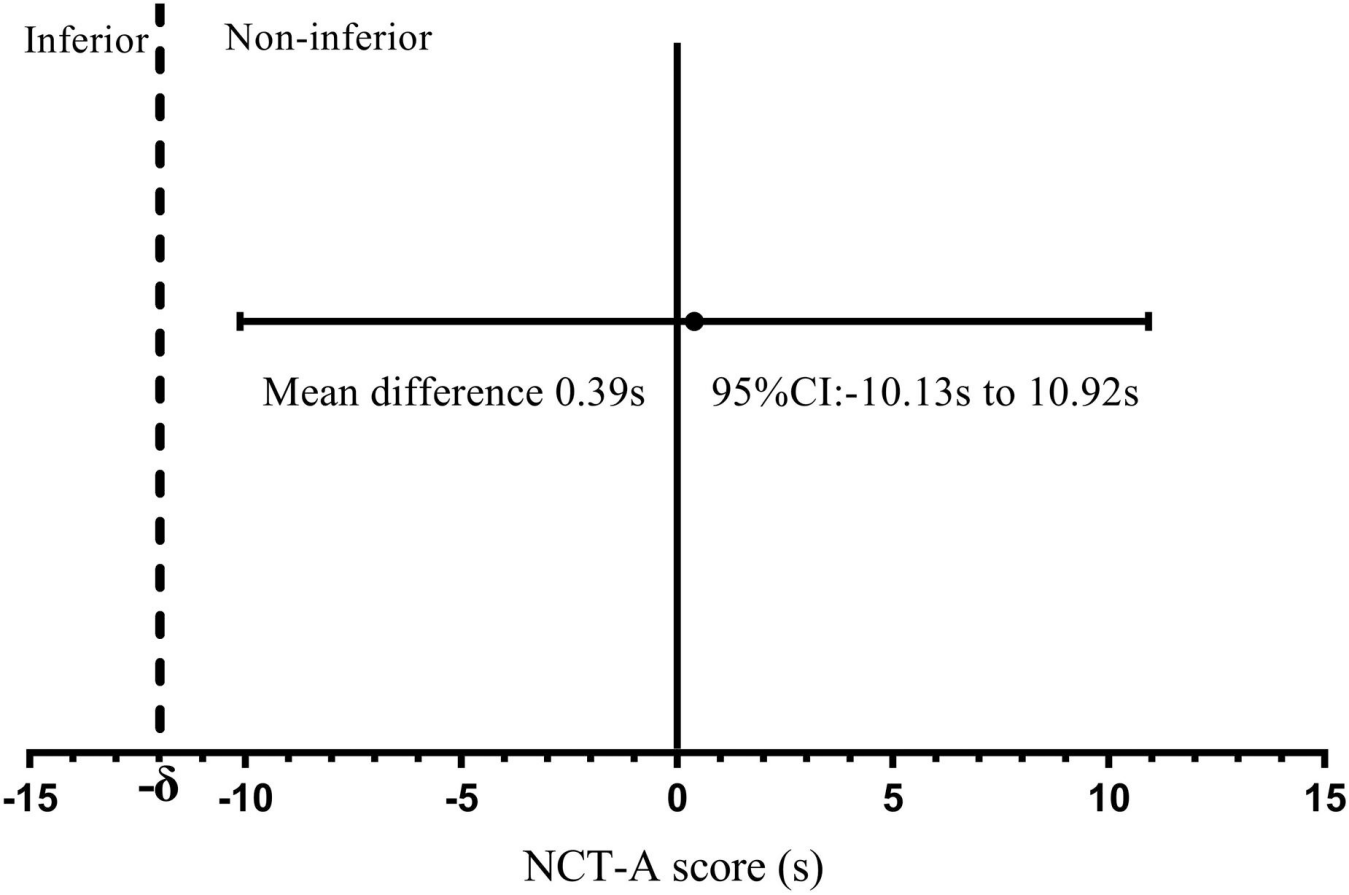
Mean difference in NCT-A scores at 6 h post-operation between both groups. Non-inferiority was assessed using confidence interval testing. The location of the effect estimate to the right of the dotted line demonstrates that group R was non-inferior to group P regarding NCT-A scores. Error bars represent the 95% confidence interval (CI).

The mean difference in NCT-A scores between the two groups at 6 h post-operation was 0.39 (95% CI: −10.13 to 10.92). The NCT-A score at post-6 h were 60.10 ± 30.05 s in group R, 59.71 ± 24.62 s in group P (*P* = 0.47). The non-inferiority margin was set at 11.94 (20% amplitude in group P of the NCT-A score at 6 h post-operation), and the lower limit of the 95% CI was greater than the −*δ* (Figure 3).

### 3.3 Secondary outcome measures

#### 3.3.1 Comparison of postoperative recovery times between the two groups

Group R demonstrated significantly shorter times to spontaneous respiration, eye opening, response to verbal commands, and extubation compared with Group P (*P* < 0.01; Table 2).

**Table 2:** Comparisons of recovery times between the two groups.

Time (s)	Group P (*n* = 54)	Group R (*n* = 54)	*P-*value
Spontaneous respiration	198.85 ± 123.44	134.63 ± 38.10	0.001★★
Eye opening	470.02 ± 220.46	185.98 ± 48.89	0.000★★
Commanded response	500.00 ± 227.63	209.55 ± 46.90	0.000★★
Extubation	512.02± 158.13	219.75 ± 60.90	0.000★★

Variables presented as mean ± standard deviation [(x¯) ± s]. Recovery times including spontaneous respiration, eye opening, commanded response, and extubation were significantly shorter in Group R than in Group P (★★*P* < 0.01).

#### 3.3.2 Modified MOAA/s scores

The modified MOAA/s score at T0 was significantly higher in Group R compared to Group P (*P* < 0.05). In both groups, the modified MOAA/s scores at T1 through T4 were significantly higher than those observed at T0 (*P* < 0.05) (Table 3).

**Table 3:** Modified MOAA/s and Steward scores at extubation and post-extubation time points.

Time point	Group P (*n* = 54)	Group R (*n* = 54)	*P-*value
Modified MOAA/s score [M(P25, P75)]			
T0	4.00 (3.00, 4.00)	4.00 (4.00, 5.00)	0.042★
T1	5.00 (4.00, 5.00)	5.00 (5.00, 5.00)	0.335
T2	5.00 (5.00, 5.00)	5.00 (5.00, 5.00)	0.724
T3	5.00 (5.00, 5.00)	5.00 (5.00, 5.00)	0.425
T4	5.00 (5.00, 5.00)	5.00 (5.00, 5.00)	0.159
Modified Steward score [M (P25, P75)]			
T0	1.00 (1.00, 1.00)	2.00 (1.00, 2.00)	0.003★★
T1	2.00 (1.00, 2.00)	2.00 (2.00, 2.00)	0.384
T2	2.00 (2.00, 2.00)	2.00 (2.00, 2.00)	0.567
T3	2.00 (2.00, 2.00)	2.00 (2.00, 2.00)	0.326
T4	2.00 (2.00, 2.00)	2.00 (2.00, 2.00)	0.326

Variables presented as median (P25, P75). Modified MOAA/s and Steward scores at extubation (T0) were significantly higher in Group R (★ *P* < 0.05, ★★ *P* < 0.01). MOAA/s, modified observer's assessment of alertness/sedation.

#### 3.3.3 Modified steward scores

At time T0, the Steward scores in Group R were significantly higher than those in Group P (*P* < 0.01). No statistically significant differences in these scores were observed between the two groups at time points T1 to T4 (*P* > 0.05). The Steward scores at T1 to T4 were significantly higher than those at T0 in both groups (*P* < 0.05).

Within 20 min after extubation, both the modified MOAA/s scores and modified Steward scores in the two groups showed a gradual increase. Ultimately, all patients reached a state of complete consciousness, clear airway, and the ability to perform purposeful limb movements (Table 3).

## 4 Discussion

In this randomized controlled trial of cirrhotic patients undergoing endoscopic variceal ligation or sclerotherapy, we compared the effects of remimazolam tosilate combined with flumazenil vs. propofol on early postoperative neurocognitive function and recovery quality. The primary neuropsychological assessments (NCT-A and DST) showed no statistically significant differences between the two groups at baseline, 6 h postoperatively, or 24 h postoperatively. However, compared to the propofol group, the remimazolam-flumazenil group demonstrated significantly shorter times to recovery of spontaneous respiration, eye opening, response to commands, and extubation, along with higher modified MOAA/S and Steward scores at the time of extubation.

In cirrhotic patients undergoing endoscopic variceal ligation or sclerotherapy, the administration of remimazolam tosylate in combination with flumazenil demonstrated comparable postoperative neurocognitive performance to propofol, without causing additional progressive neurocognitive impairment. Moreover, this regimen significantly shortened the time to recovery of spontaneous breathing, awakening, response to commands, and extubation, while also improving the quality of awakening immediately after extubation. Consequently, this agent presents a sedative-anesthetic option characterized by a neurocognitive profile comparable to propofol and a more rapid, controllable recovery profile for this high-risk population.

The NCT-A and DST were employed as the primary assessment instruments, owing to their established validity as sensitive and specific tools for screening minimal hepatic encephalopathy (MHE) and detecting subtle perioperative neurocognitive changes (23). Analysis revealed no statistically significant intergroup differences in the changes (delta values) of neuropsychological scores at preoperative, 6 h postoperative, and 24 h postoperative time points. Furthermore, confidence interval analysis demonstrated that remimazolam was non-inferior to propofol with respect to the NCT-A score at 6 h postoperatively. Notably, both groups exhibited a significant reduction in NCT-A completion time and a significant increase in DST scores at 24 h postoperatively compared to baseline levels. These findings indicate that remimazolam tosilate and propofol do not induce acute progressive central nervous system impairment or increase the risk of hepatic encephalopathy when utilized for anesthesia in cirrhotic patients undergoing EVL and/or EVS, demonstrating comparable neurological safety profiles. The observed improvement in postoperative neuropsychological scores may be attributed to the mitigation of bleeding risk and partial alleviation of portal pressure following successful endoscopic intervention, as well as reduced postoperative pain and stress, which may facilitate the recovery of attention and executive function. Nevertheless, the potential influence of a “practice effect” resulting from repeated testing cannot be entirely excluded (22). It is noteworthy that the remimazolam group received routine antagonism with flumazenil upon the conclusion of the procedure. As a specific competitive antagonist of benzodiazepine receptors, flumazenil rapidly reverses the sedative effects of remimazolam (18). Previous studies have indicated that flumazenil antagonism can reduce the incidence of post-endoscopic hepatic encephalopathy in cirrhotic patients (19). Consequently, the combined strategy of remimazolam and flumazenil adopted in this study not only leverages the hepatic metabolism-independent property of remimazolam but also ensures the rapid clearance of residual sedation through specific pharmacological reversal. This mechanism may constitute the key pharmacological basis for the favorable neurological safety profile observed.

Recovery quality is a key evaluation aspect in perioperative anesthesia management for cirrhotic patients undergoing endoscopic procedures (24). Rapid and smooth recovery helps reduce fluctuations in respiratory and circulatory functions and may decrease the risk of related complications. The secondary outcome measures of this study demonstrated that, compared to the propofol group, the remimazolam group had significantly shorter times to recovery of spontaneous breathing, awakening, response to commands, and extubation. Additionally, at the time of extubation (T0), the modified MOAA/s scores and modified Steward scores were significantly higher in the remimazolam group, indicating that remimazolam combined with flumazenil promotes faster and higher-quality early recovery. The core mechanisms underlying this accelerated and improved recovery are the rapid, non-hepatic metabolism of remimazolam without intraoperative accumulation, coupled with the immediate and effective reversal provided by flumazenil. A meta-analysis also supports that, compared to propofol, remimazolam combined with flumazenil can accelerate patient awakening, shorten extubation time, and reduce PACU stay duration (25). No statistically significant differences in recovery scores were observed between the two groups from 5 to 20 min following extubation, suggesting that propofol facilitates a satisfactory gradual recovery; however, the remimazolam-based regimen demonstrated a more distinct advantage in early recovery. Clinically, faster awakening and extubation can shorten the length of stay in the PACU and improve the efficiency of medical resource utilization. A smoother recovery process also helps reduce adverse events such as agitation, nausea, and vomiting, thereby enhancing patient comfort and safety (25). While prior literature associates flumazenil reversal after prolonged remimazolam infusion with resedation risk (26), our study focused on short-duration procedures (mean 28 min) with low remimazolam doses and rapid metabolism. In this setting, standardized flumazenil reversal resulted in stable recovery without resedation or respiratory re-depression. This suggests the remimazolam-flumazenil regimen is safe and reliable for short surgeries, with risks likely confined to prolonged-infusion scenarios.

This study has several limitations. First, limited by the single-center design and operational conditions, this study had a relatively small sample size and failed to investigate the effects of sevoflurane on postoperative neurological function and quality of recovery in cirrhotic patients undergoing endoscopic treatment. Nevertheless, this study strictly adhered to the principles of randomized controlled trials, and the study protocol remained unchanged. Second, Our study used awake endotracheal intubation for cirrhotic patients, differing from the deep sedation/conventional induction common elsewhere. This technique may affect sympathetic tone, stress hormones, and recovery (27), limiting generalizability to alternative airway settings. However, the comparison between remimazolam-flumazenil and propofol remains robust, as identical airway management minimized confounding in between-group analyses. Third, missing pre-6-hour assessments may have overlooked transient acute drug effects that resolved early, limiting our ability to capture short-term pharmacodynamic changes. Future studies should add frequent early monitoring. Additionally, the 24 h postoperative window excluded long-term follow-up on neuropsychological function, hepatic encephalopathy recurrence, or prognosis (7 days, 1 month, or later). Thus, long-term safety and efficacy of the regimens remain unclear, requiring extended follow-up trials. Fourth, NCT-A and DST assess only attention, executive function, and visuomotor coordination, limiting their ability to comprehensively evaluate neurological function. Consequently, these findings should be interpreted as reflecting short-term postoperative cognitive performance within the context of minimal hepatic encephalopathy screening, rather than constituting a definitive evaluation of overall neurological safety. Additionally, the study relied solely on neuropsychological scales, lacking objective measures (EEG, blood ammonia, neurological injury biomarkers), indicating room to expand evaluation dimensions. Finally, subgroup analyses based on Child-Pugh classification were not performed; differences in responses to remimazolam vs. propofol among patients with varying hepatic functional reserves remain to be explored. Despite the aforementioned limitations, this study still provides preliminary evidence for the selection of anesthesia in endoscopic treatment for patients with liver cirrhosis, suggesting that intravenous anesthesia may be a safer alternative.

In cirrhotic patients undergoing EVL and/or EVS, the administration of remimazolam tosilate combined with flumazenil demonstrated early postoperative neurocognitive performance comparable to that of propofol, as assessed by NCT-A and DST at 6 and 24 h postoperatively. Additionally, this regimen significantly shortened recovery and extubation times and enhanced the immediate quality of awakening. These findings suggest that the remimazolam-flumazenil combination represents a viable anesthetic option for this patient population, offering advantages in terms of early recovery. However, the long-term neurocognitive safety of this regimen remains to be established, and our results should be interpreted within the context of the study's limitations, including the short follow-up period, single-center design, and the use of neuropsychological tests that assess specific cognitive domains rather than comprehensive neurological safety.

## Data Availability

The original contributions presented in the study are included in the article/Supplementary Material, further inquiries can be directed to the corresponding author/s.

## References

[1] Pallio S Melita G Shahini E Vitello A Sinagra E Lattanzi B. Diagnosis and management of esophagogastric varices. Diagnostics (Basel). (2023) 13:1031. 10.3390/diagnostics13061031 36980343 PMC10047815

[2] Mansour L El-Kalla F El-Bassat H Abd-Elsalam S El-Bedewy M Kobtan A. Randomized controlled trial of scleroligation versus band ligation alone for eradication of gastroesophageal varices. Gastrointest Endosc. (2017) 86:307–15. 10.1016/j.gie.2016.12.026 28082116

[3] Wu W Liu Q Li W Kan J Wang Q Wang L. Ligation-occluded endoscopic injection sclerotherapy: a novel retrograde strategy for gastroesophageal varices obliteration. Endoscopy. (2021) 53:E328–9. 10.1055/a-1275-9674 33096574

[4] Shi F Chen Y Li H Zhang Y Zhao T. Efficacy and safety of remimazolam tosilate versus propofol for general anesthesia in cirrhotic patients undergoing endoscopic variceal ligation. Int J Gen Med. (2022) 15:583–91. 10.2147/IJGM.S345390 35046716 PMC8763269

[5] Mullen KD Prakash RK. Management of covert hepatic encephalopathy. Clin Liver Dis. (2012) 16:91–3. 10.1016/j.cld.2011.12.006 22321467

[6] Ginès P Krag A Abraldes JG Solà E Fabrellas N Kamath PS. Liver cirrhosis. Lancet. (2021) 398:1359–76. 10.1016/S0140-6736(21)01374-X 34543610

[7] Reves JG Fragen RJ Vinik HR Greenblatt DJ. Midazolam: pharmacology and uses. Anesthesiology. (1985) 62:310–24. 10.1097/00000542-198503000-00017 3156545

[8] Jeong J Jung K Seo KI Yun BC Han BH Lee SU. Propofol alone prevents worsening hepatic encephalopathy rather than midazolam alone or combined sedation after esophagogastroduodenoscopy in compensated or decompensated cirrhotic patients. Eur J Gastroenterol Hepatol. (2020) 32:1054–61. 10.1097/MEG.0000000000001755 32433420 PMC7337114

[9] Wahab EA Hamed EF Ahmad HS Abdel Monem SM Fathy T. Conscious sedation using propofol versus midazolam in cirrhotic patients during upper GI endoscopy: a comparative study. JGH Open. (2019) 3:25–31. 10.1002/jgh3.12098 30834337 PMC6386741

[10] Redfield R Latt N Munoz SJ. Minimal hepatic encephalopathy. Clin Liver Dis. (2024) 28:237–52. 10.1016/j.cld.2024.01.004 38548436

[11] Duarte-Rojo A Allampati S Thacker LR Flud CR Patidar KR White MB. Diagnosis of covert hepatic encephalopathy: a multi-center study testing the utility of single versus combined testing. Metab Brain Dis. (2019) 34:289–95. 10.1007/s11011-018-0350-z 30506333 PMC6351159

[12] Zhang P Gan D Chi X Mao D Gao Y Li Y. Regression-based Chinese norms of number connection test A and digit symbol test for diagnosing minimal hepatic encephalopathy. Sci Rep. (2024) 14:4005. 10.1038/s41598-024-54696-4 38369632 PMC10874952

[13] Khamaysi I William N Olga A Alex I Vladimir M Kamal D. Sub-clinical hepatic encephalopathy in cirrhotic patients is not aggravated by sedation with propofol compared to midazolam: a randomized controlled study. J Hepatol. (2011) 54:72–7. 10.1016/j.jhep.2010.06.023 20934771

[14] Amorós A Aparicio JR Garmendia M Casellas JA Martínez J Jover R. Deep sedation with propofol does not precipitate hepatic encephalopathy during elective upper endoscopy. Gastrointest Endosc. (2009) 70:262–8. 10.1016/j.gie.2008.10.038 19394004

[15] Wadhwa V Issa D Garg S Lopez R Sanaka MR Vargo JJ. Similar risk of cardiopulmonary adverse events between propofol and traditional anesthesia for gastrointestinal endoscopy: a systematic review and meta-analysis. Clin Gastroenterol H. (2017) 15:194–206. 10.1016/j.cgh.2016.07.013 27451091

[16] Ziser A Plevak DJ. Morbidity and mortality in cirrhotic patients undergoing anesthesia and surgery. Curr Opin Anaesthesiol. (2001) 14:707–11. 10.1097/00001503-200112000-00018 17019169

[17] Lee A Shirley M. Remimazolam: a review in procedural sedation. Drugs. (2021) 81:1193–201. 10.1007/s40265-021-01544-8 34196946

[18] Sato T Nishiwaki K. Remimazolam should be antagonized by an adequate flumazenil. J Anesth. (2023) 37:164–65. 10.1007/s00540-022-03117-9 36197518

[19] Kim SI Jin YJ Lee SH Lee JW Lee DH Lee D. Conscious sedation using midazolam and sequential flumazenil in cirrhotic patients for prophylactic endoscopic variceal ligation. Digestion. (2015) 92:220–6. 10.1159/000440715 26488160

[20] Shi F Tang R Du X Li X Wu G. Application of remimazolam-0.6% sevoflurane anesthesia for flash visual evoked potential monitoring during pituitary adenoma resection: a non-inferiority randomized controlled trial. BMC Anesthesiol. (2024) 24:85. 10.1186/s12871-024-02466-0 38424486 PMC10903035

[21] Stöhr T Colin PJ Ossig J Pesic M Borkett K Winkle P. Pharmacokinetic properties of remimazolam in subjects with hepatic or renal impairment. Br J Anaesth. (2021) 127:415–23. 10.1016/j.bja.2021.05.027 34246461

[22] Weissenborn K Ennen JC Schomerus H Rückert N Hecker H. Neuropsychological characterization of hepatic encephalopathy. J Hepatol. (2001) 34:768–73. 10.1016/s0168-8278(01)00026-5 11434627

[23] Ji J Liu YY Wu GW Hu YL Liang CH Wang XD. Changes in dynamic and static brain fluctuation distinguish minimal hepatic encephalopathy and cirrhosis patients and predict the severity of liver damage. Front Neurosci. (2023) 17:1077808. 10.3389/fnins.2023.1077808 37056312 PMC10086246

[24] Lyckner S Chew MS Nilsson A. Quality of recovery and pre-existing impaired cognition in patients undergoing advanced GI endoscopic procedures with patient-controlled sedation: a prospective observational cohort study. IGIE. (2023) 2:292–98.e5. 10.1016/j.igie.2023.07.002 41646995 PMC12851060

[25] Wu Q Xu F Wang J Jiang M. Comparison of remimazolam-flumazenil versus propofol for recovery from general anesthesia: a systematic review and meta-analysis. J Clin Med. (2023) 12:7316. 10.3390/jcm12237316 38068368 PMC10707179

[26] Masui K. Caution!! Reappearance of remimazolam effect after a flumazenil bolus: a larger bolus of flumazenil and a lower total remimazolam clearance are higher risks. J Anesth. (2023) 37:1–5. 10.1007/s00540-022-03107-x 36114320

[27] Abd Elhafez NF Oriquat GA Nsairat H Jaber B Abdelraouf AM Fahmy MK. RETRACTED: interaction between intubation and stress regarding hemodynamic and hormonal changes. J Int Med Res. (2026) 54:3000605261437457. 10.1177/03000605261437457 39939114 PMC11822838

